# Impact of concomitant mitral valve surgery on the clinical outcomes of patients with moderate functional mitral regurgitation and HFpEF undergoing aortic valve replacement: a cohort study

**DOI:** 10.1186/s13019-023-02197-2

**Published:** 2023-04-05

**Authors:** Xieraili Tiemuerniyazi, Ziang Yang, Yifeng Nan, Yangwu Song, Wei Zhao, Fei Xu, Wei Feng

**Affiliations:** 1 Fuwai Hospital, National Center for Cardiovascular Diseases, National Clinical Research Center for Cardiovascular Diseases, Chinese Academy of Medical Sciences and Peking Union Medical College, Beilishi Road No. 167, Xicheng District, Beijing, 100037 China; 2 Department of Cardiovascular Surgery, Yunnan Fuwai Cardiovascular Hospital, Kunming, China

**Keywords:** Functional mitral regurgitation, Heart failure with preserved ejection fraction, Aortic valve replacement, Mitral valve surgery

## Abstract

**Background:**

Functional mitral regurgitation (FMR) worsens the prognosis of patients with heart failure with preserved ejection fraction (HFpEF). While concomitant mitral valve surgery (MVS) is recommended for severe FMR during aortic valve replacement (AVR), the optimal treatment of moderate FMR, especially in those with HFpEF, remains unclear. This study aimed to evaluate the effect of MVS in patients with moderate FMR and HFpEF undergoing AVR.

**Methods:**

A total of 212 consecutive patients (AVR: 34.0%, AVR-MVS: 66.0%) during 2010 and 2019 were enrolled. Survival outcomes were compared. Inverse probability treatment weighting (IPTW) was used to balance the baseline characteristics. Kaplan-Meier curve and log-rank test were applied to compare the survival outcomes. The primary endpoint was the overall mortality.

**Results:**

The mean age was 58.9 $\pm$ 11.9 years, and 27.8% of them were female. During a median follow-up of 16.4 months, AVR-MVS did not reduce the risk of mid-term MACCE (hazard ratio [HR]: 1.53, 95% confidence interval [CI]: 0.57–4.17, P_log-rank_ = 0.396), while it showed a tendency toward higher MACCE risk in the IPTW analysis (HR: 2.62, 95% CI: 0.84–8.16, P_log-rank_ = 0.096). In addition, AVR-MVS increased the risk of mortality as compared to isolated AVR (0 vs. 10%, P_log-rank_ = 0.016), which was sustained in the IPTW analysis  (0 vs. 9.9%, P_log-rank_<0.001).

**Conclusion:**

In patients with moderate FMR and HFpEF, isolated AVR might be more reasonable than AVR-MVS.

**Supplementary Information:**

The online version contains supplementary material available at 10.1186/s13019-023-02197-2.

## Introduction

Heart failure with preserved ejection fraction (HFpEF) stands for heart failure with symptoms and signs without abnormality in ejection fraction (EF), and accounts for more than half of the heart failure [1]. Functional mitral regurgitation (FMR) is common in patients with heart failure. Previous studies demonstrate that FMR can compromise the prognosis of patients in chronic heart failure patients with reduced ejection fraction [2]. In recent years, researchers also notice that even mild FMR is associated with increased risk of adverse events in HFpEF patients [3], whereas mitral valve repair improves clinical outcomes [4, 5]. Aortic valve disease is one of the major causes of FMR and heart failure. A substantial number of patients undergoing aortic valve replacement (AVR) can be complicated with FMR and HFpEF in the clinical practice. While concomitant mitral valve surgery (MVS) is recommended in patients with severe FMR undergoing AVR [6], treatment of moderate FMR is controversial [7, 8]. Limited data is available. The aim of this study was to examine the effect of isolated AVR and AVR-MVS on the prognosis of HFpEF patients complicated with severe aortic valve disease and moderate FMR.

## Methods

This is a retrospective cohort analysis of consecutive HFpEF and moderate FMR patients referred to our center for surgical AVR procedure between January 2010 and December 2019. Two different surgical strategies, namely isolated AVR and AVR-MVS, were compared. The investigation conforms with the principles outlined in the *Declaration of Helsinki* [9]*.* The Institutional Review Board at our institute approved the use of clinical data for this study (*NO.: 2021-1585*) and waived individual informed consent.

Patients who fulfilled all of the following criteria were included: (1) > 18 years of age; (2) underwent AVR for severe aortic valve disease; and (3) complicated with moderate FMR and HFpEF. Patients with (1) rheumatic valvular heart disease; (2) a history of infective endocarditis; (3) a primary lesion on mitral leaflets, papillary muscle or chordae tendineae; or (4) incomplete clinical data were excluded.

The primary outcome was the overall mortality. The secondary outcomes were as follows: perioperative complications, the improvement of FMR, EF, left atrial diameter (LAD), left ventricular end-diastolic diamete (LVEDD)r, and major adverse cardiovascular and cerebrovascular events (MACCE), which was defined as the composite of all-cause death, myocardial infarction, ischemic or hemorrhagic stroke, hospitalization for heart failure and repeat valvular surgery.

FMR was defined as mitral regurgitation without evidence of primary lesion on mitral valve leaflets, papillary muscle or chordae tendineae, and was determined using transthoracic echocardiography at least for twice preoperatively. FMR was divided into five entities according to the vena contracta and regurgitant jet area, namely 0^+^  = no, 1^+^  = trace, 2^+^  = mild, 3^+^  = moderate, 4^+^  = severe, and only patient with 3^+^ were enrolled. Transesophageal echocardiography was performed to further evaluation of the FMR in the operating room before the surgery. Improvement of FMR was defined as the decrease of regurgitation for at least one level. The definition of HFpEF was in line with the clinical guidelines [10], which was diagnosed according to the presence of heart failure symptoms and/or signs, elevated N-terminal pro-B type natriuretic peptide (NT-proBNP, > 125 pg/mL), and normal EF ($\ge 50\mathrm{\%}$).

Other definitions were as follows: operative death was defined as death within 30 days after surgery; postoperative acute kidney failure was defined according to Kidney Disease Improving Global Guidelines criteria [11].

Electronic hospital records were used to extract the baseline and perioperative data, while follow-up data were collected from outpatient routine check-up. For those who were unavailable for re-examination at our institute, phone call interviews were used to complete the follow-up.

The median thoracotomy was applied, and all of the procedures were performed using cardiopulmonary bypass. Since there’s no standard treatment for moderate FMR, whether to perform concomitant mitral valve surgery, as well as the selection of mitral valve repair (MVr) or replacement (MVR), was decided by the surgeons. Generally, surgeons might tend to perform MVS for those with large left ventricles or mitral annulus, eccentric mitral regurgitation and/or very longstanding course of aortic valve disease. For those who were assessed by the surgeons to be at higher risk of FMR recurrence, MVR was performed.

Continuous variables were presented as mean ± standard deviation and tested by Student t-test if normally distributed. Otherwise, they were presented as medians with the 25th and 75th percentiles and tested by rank-sum test. Categorical variables were presented as numbers (%) and tested by Chi-square test or Fisher exact test, as appropriate. Survivals were calculated with the Kaplan–Meier method and compared by the log-rank test. Inverse probability treatment weighting (IPTW) analysis was performed to balance the baseline characteristics of the patients. In the IPTW analysis, balanced preoperative variables were as follows: age, sex, body mass index, body surface area, atrial fibrillation, hypertension, dyslipidemia, coronary artery disease, New York Heart Association (NYHA) class III or IV, diabetes mellitus, renal failure, EF, LAD, LVEDD, type of aortic valve disease, type of aortic prosthesis, and concomitant procedure such as coronary artery bypass grafting, tricuspid valve repair and other surgeries. Variables with a standardized mean difference < 0.2 or P-value > 0.05 was considered to be well-balanced. A P value < 0.05 was considered statistically significant. Statistical analyses were performed using R 4.1.2 (R Core Team, Vienna, Austria).

## Results

Of the 212 patients with moderate FMR and HFpEF, 34.0% underwent isolated AVR, and 66.0% underwent AVR-MVS. More than half of the AVR-MVS patients (55.0%) received MVr, while the remainders received MVR. The mean age was 58.9 $\pm$ 11.9 years in the whole cohort, and 27.8% of them were female. Compared with AVR, subjects in AVR-MVS were younger, more likely to have aortic insufficiency rather than aortic stenosis and more complicated with prior stroke. In addition, AVR-MVS patients also shared larger LAD, LVEDD and lower EF, while the levels of preoperative NT-proBNP were comparable between the two groups. What’s more, patients in the AVR-MVS also displayed more chance of receiving tricuspid valve surgery (Table 1).

**Table 1:** Preoperative and intraoperative characteristics

Variables	Original			ITPW analysis		
	AVR (N = 72)	AVR-MVS (N = 140)	P value	AVR (N = 216.03)	AVR-MVS (N = 207.63)	P value
*Preoperative*						
Age (years), mean ± SD	61.3 ± 10.5	57.7 ± 12.4	0.039	58.2 ± 11.2	58.5 ± 11.9	0.903
Female, no (%)	25 (34.7)	34 (24.3)	0.108	89.6 (41.5)	55.8 (26.9)	0.208
BMI (kg/m^2^), median [Q1, Q3]	23.5 [20.1, 26.3]	23.5 [21.5, 26.2]	0.312	22.2 [20.4, 25.3]	23.3 [21.5, 26.1]	0.262
BSA (m^2^), median [Q1, Q3]	1.6 [1.8, 1.9]	1.6 [1.8, 1.9]	0.566	1.7 [1.5, 1.9]	1.8 [1.7, 1.9]	0.280
Hypertension, no (%)	37 (51.4)	56 (40.0)	0.114	123.4 (57.1)	90.5 (43.6)	0.208
Dyslipidemia, no (%)	25 (34.7)	49 (35.0)	0.968	72.6 (33.6)	76.9 (37.0)	0.733
Smoking, No. (%)	29 (40.3)	62 (44.3)	0.577	77.3 (35.8)	88.8 (42.8)	0.483
Diabetes mellitus, no (%)	10 (13.9)	13 (9.3)	0.307	17.2 (7.9)	20.3 (9.8)	0.657
Coronary artery disease, no (%)	10 (13.9)	25 (17.9)	0.461	27.1 (12.5)	34.4 (16.6)	0.492
Atrial fibrillation, no (%)	9 (12.5)	23 (16.4)	0.449	61.4 (28.4)	33.0 (15.9)	0.279
Renal failure, no (%)	1 (1.4)	8 (5.7)	0.279	4.5 (2.1)	9.0 (4.3)	0.479
Stroke, no (%)	10 (13.9)	6 (4.3)	0.012	13.6 (6.3)	10.2 (4.9)	0.652
Aortic valve disease, no (%)			0.017			0.370
Aortic insufficiency, no (%)	39 (54.2)	99 (70.7)		126.3 (58.5)	143.0 (68.9)	
Aortic stenosis	33 (45.8)	41 (29.3)		89.7 (41.5)	64.6 (31.1)	
NYHA III/IV, no (%)	33 (45.8)	74 (52.9)	0.333	128.1 (59.3)	109.6 (52.8)	0.532
LAD (mm), mean ± SD	42.2 ± 6.2	46.4 ± 6.3	< 0.001	44.6 ± 6.1	45.3 ± 6.3	0.547
LVEDD (mm), mean ± SD	59.4 ± 10.3	64.9 ± 9.0	< 0.001	63.7 ± 10.2	63.6 ± 9.0	0.942
EF (%), mean ± SD	59.7 ± 5.5	58.2 ± 4.8	0.037	59.0 ± 4.9	58.7 ± 4.8	0.701
NT-proBNP (pg/ml), median [Q1, Q3]	954.6 [500.7, 2282.5]	1404.0 [731.0, 2474.9]	0.058	1436.1 [591.0, 2193.9]	1300.0 [631.3, 2373.2]	0.903
*Operative*						
CPB (min), median [Q1, Q3]	106.0 [82.0, 133.5]	145.0 [123.0, 183.0]	< 0.001	116.3 [83.2, 154.0]	146.0 [125.0, 187.5]	0.073
Cross-clamp (min), median [Q1, Q3]	77.5 [57.5, 104.0]	112.5 [92.0, 139.5]	< 0.001	95.0 [59.7, 126.5]	115.5 [92.0, 141.6]	0.112
TV repair, no (%)	3 (4.2)	44 (31.4)	< 0.001	51.0 (23.6)	47.0 (22.6)	0.942
CABG, no (%)	9 (12.5)	19 (13.6)	0.827	21.8 (10.1)	27.3 (13.1)	0.545
Other procedures, no (%)	8 (11.1)	14 (10.0)	0.802	23.3 (10.8)	20.5 (9.9)	0.867
Perioperative IABP, no (%)	0	1 (0.7)	> 0.99	0	1.4 (0.7)	0.312

AVR, aortic valve replacement; BMI, body mass index; BSA, body surface area; CABG, coronary artery bypass grafting; CPB, cardiopulmonary bypass; EF, ejection fraction; IABP, intra-aortic balloon pump; IPTW, inverse probability treatment weighting; LAD, left atrial diameter; LVEDD, left ventricular end-diastolic diameter; MVS, mitral valve surgery; NT-proBNP, N-terminal pro-B type natriuretic peptide; NYHA, New York Heart Association; SD, standard deviation; TV, tricuspid valve

Since differences existed in the baseline characteristics of the patients, subsequent results were presented before and after adjustment. The total number of patients in the IPTW analysis was 423.66, and all of the baseline and operative confounders were considered to be well-balanced (Table 1).

As compared to AVR, AVR-MVS increased the duration of cardiopulmonary bypass (P < 0.001), as well as the cross-clamp time (P < 0.001) in the unmatched cohort, but not in the IPTW analysis. In the IPTW analysis, AVR-MVS was observed to be associated with increased risk of reoperation for bleeding (0 vs. 4.0%, P = 0.034), operative mortality (0 vs. 3.1%, P = 0.031), and less reduction in the size of LAD (− 8.4 ± 4.6 mm vs. − 6.5 ± 6.3 mm, P = 0.038), while the improvement rate of FMR was comparable between the two groups (100% vs. 100%) (Table 2).

**Table 2:** Early postoperative and follow-up outcomes

Variables	Original			IPTW analysis		
	AVR (N = 72)	AVR-MVS (N = 140)	P value	AVR (N = 216.03)	AVR-MVS (N = 207.63)	P value
*Early postoperative*						
Perioperative transfusion, no (%)	18 (25.0)	16 (11.4)	0.011	49.1 (22.7)	23.6 (11.4)	0.085
New-onset stroke, no (%)	0	1 (0.71)	> 0.99	0	1.0 (0.5)	0.313
New-onset atrial fibrillation, no (%)	2 (2.8)	9 (6.4)	0.256	6.3 (2.9)	17.1 (8.2)	0.223
Acute kidney injury, no (%)	8 (11.1)	11 (7.9)	0.453	18.4 (8.5)	15.6 (7.5)	0.810
Thoracotomy for bleeding, no (%)	0	5 (3.6)	0.169	0	8.2 (4.0)	0.034
Operative death, no (%)	0	5 (3.6)	0.169	0	6.4 (3.1)	0.031
LAD (mm), mean ± SD	35.2 ± 4.8	39.2 ± 5.6	< 0.001	36.2 ± 4.6	38.7 ± 5.6	0.003
ΔLAD (mm), mean ± SD	− 7.0 ± 4.7	− 7.2 ± 6.3	0.813	− 8.4 ± 4.6	− 6.5 ± 6.3	0.038
LVEDD (mm), mean ± SD	50.0 ± 7.4	53.6 ± 7.2	< 0.001	52.0 ± 6.6	52.9 ± 7.5	0.485
ΔLVEDD (mm), mean ± SD	− 9.5 ± 6.3	− 11.3 ± 7.5	0.078	− 11.8 ± 6.7	− 10.7 ± 7.4	0.441
EF (%), mean ± SD	56.2 ± 8.2	52.4 ± 8.3	0.002	53.7 ± 8.3	52.8 ± 8.5	0.647
ΔEF (%), mean ± SD	− 3.5 ± 7.9	− 5.7 ± 8.0	0.058	− 5.4 ± 7.3	− 5.9 ± 8.3	0.733
Mitral regurgitation, no (%)			0.016			0.068
No	42 (58.3)	108 (77.1)		132.6 (61.4)	159.5 (76.8)	
Trivial	28 (38.9)	29 (20.7)		80.2 (37.1)	40.0 (19.3)	
Mild	2 (2.8)	3 (2.1)		3.2 (1.5)	8.1 (3.9)	
Improvement of FMR, no (%)	72 (100)	140 (100)	> 0.99	216.0 (100)	207.6 (100)	> 0.99
*Follow-up*						
All-cause death, no (%)	0	14 (10.0)	0.016^a^	0	20.6 (9.9)	< 0.001 ^a^
MACCE, no (%)	5 (6.9)	18 (12.8)	0.396^a^	9.5 (4.4)	26.2 (12.6)	0.096 ^a^

AVR, aortic valve replacement; FMR, functional mitral regurgitation; EF, ejection fraction; IPTW, inverse probability treatment weighting; LAD, left atrial diameter; LVEDD, left ventricular end-diastolic diameter; MVS, mitral valve surgery; MACCE, major adverse cardiovascular and cerebrovascular events; SD, standard deviation

^a^Log-rank test

**Fig. 1:**
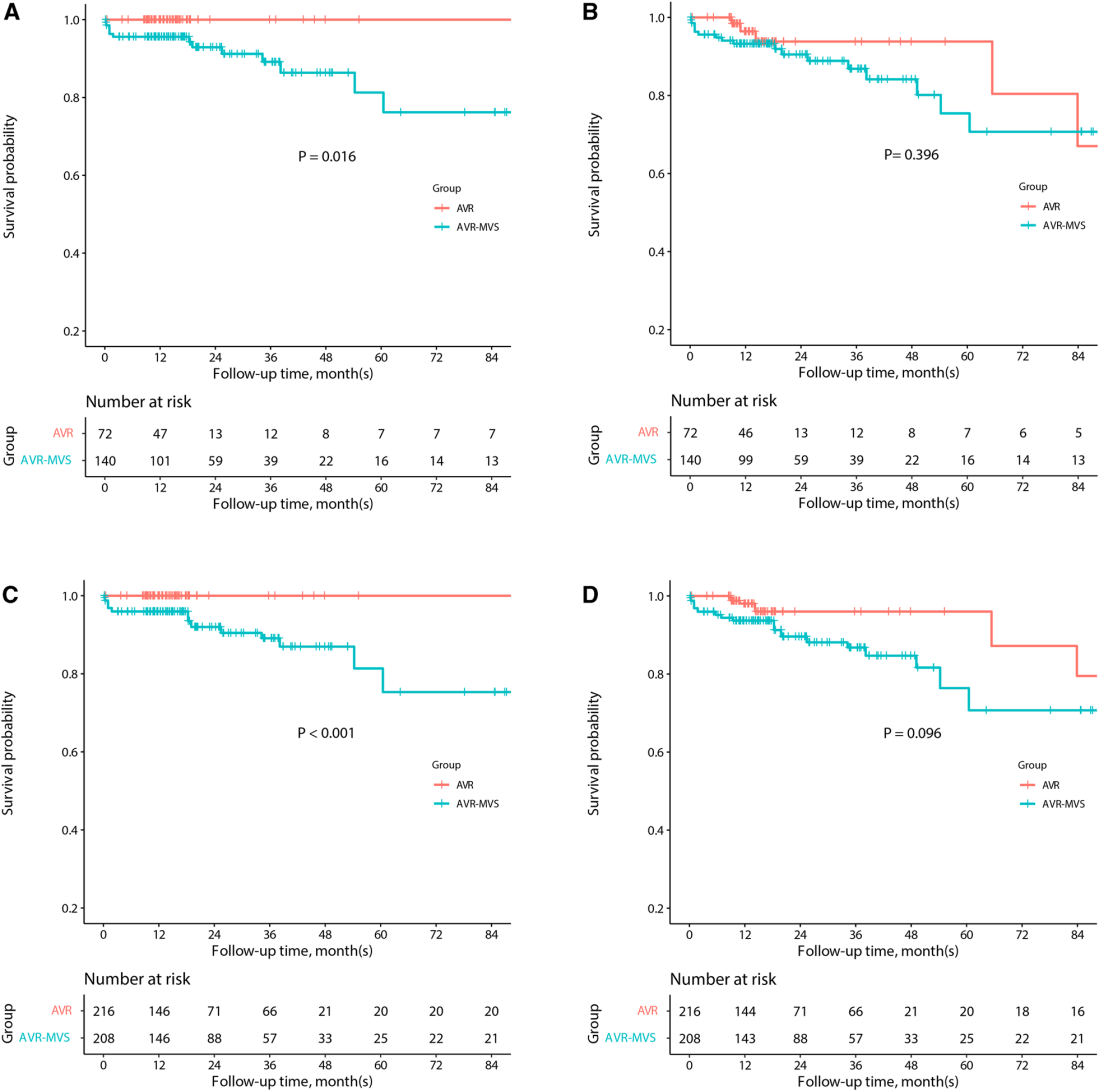
Kaplan–Meier estimates of survival outcomes in the overall cohort. Overall survivals in the original cohort (**A**) and IPTW analysis (**C**), as well as MACCE-free survivals in the original cohort (**B**) and IPTW analysis (**D**). AVR, aortic valve replacement; IPTW, inverse probability treatment weighting; MACCE, major adverse cardiovascular and cerebrovascular events; MVS, mitral valve surgery

The median follow-up time was 16.4 [10.9, 34.8] months. Fourteen patients suffered from death, including 11 from cardiac death, 2 from stroke, and 1 from traffic accident. AVR-MVS was observed to be associated with increased risk of mortality as compared to AVR (0 vs. 10%, P_log-rank_ = 0.016), which was sustained in the IPTW analysis  (0 vs. 9.9%, P_log-rank _< 0.001). In addition, AVR-MVS showed a tendency toward higher risk of MACCE (Hazard Ratio [HR]: 1.53, 95% confidence interval [CI]: 0.57–4.17, P_log-rank_ = 0.396), especially in the IPTW analysis (HR: 2.62, 95% CI: 0.84–8.16, P_log-rank_ = 0.096) (Fig. 1).

Echocardiographic results of 154 (72.6%) were available during the follow-up, most of which were finished during 3-12 months postoperatively. FMR had improved in all of the patients. AVR-MVS achieved more reduction in the LVEDD than AVR (P = 0.047), while the significance disappeared in the IPTW analysis (Table 3).

**Table 3:** Follow-up echocardiography

Variables	Original			IPTW analysis		
	AVR (N = 53)	AVR-MVS (N = 101)	P value	AVR (N = 177.83)	AVR-MVS (N = 151.63)	P value
LAD (mm), mean ± SD	38.1 ± 5.8	40.2 ± 6.5	0.055	38.1 ± 5.5	40.4 ± 6.4	0.034
ΔLAD (mm), mean ± SD	− 6.1 ± 9.9	− 7.7 ± 10.6	0.346	− 15.3 ± 16.6	− 6.0 ± 10.5	0.138
LVEDD (mm), mean ± SD	48.5 ± 5.4	51.3 ± 7.1	0.012	48.6 ± 4.4	50.2 ± 6.8	0.097
ΔLVEDD (mm), mean ± SD	− 11.5 ± 7.9	− 14.4 ± 8.9	0.047	− 15.7 ± 8.6	− 13.8 ± 8.8	0.435
EF (%), mean ± SD	60.1 ± 6.0	58.3 ± 8.5	0.163	58.2 ± 6.3	58.4 ± 8.5	0.906
ΔEF (%), mean ± SD	0.9 ± 6.7	0.3 ± 8.8	0.679	− 0.6 ± 6.1	− 0.2 ± 8.9	0.784
Mitral regurgitation, no (%)			0.059			< 0.001
No	35 (66.0)	81 (80.2)		2.4 (1.3)	4.8 (3.2)	
Trivial	17 (32.1)	16 (15.8)		80.5 (45.3)	116.8 (77.0)	
Mild	1 (1.9)	4 (4.0)		94.9 (53.4)	30.1 (19.9)	

AVR, aortic valve replacement; FMR, functional mitral regurgitation; EF, ejection fraction; IPTW, inverse probability treatment weighting; LAD, left atrial diameter; LVEDD, left ventricular end-diastolic diameter; MVS, mitral valve surgery; SD, standard deviation

**Fig. 2:**
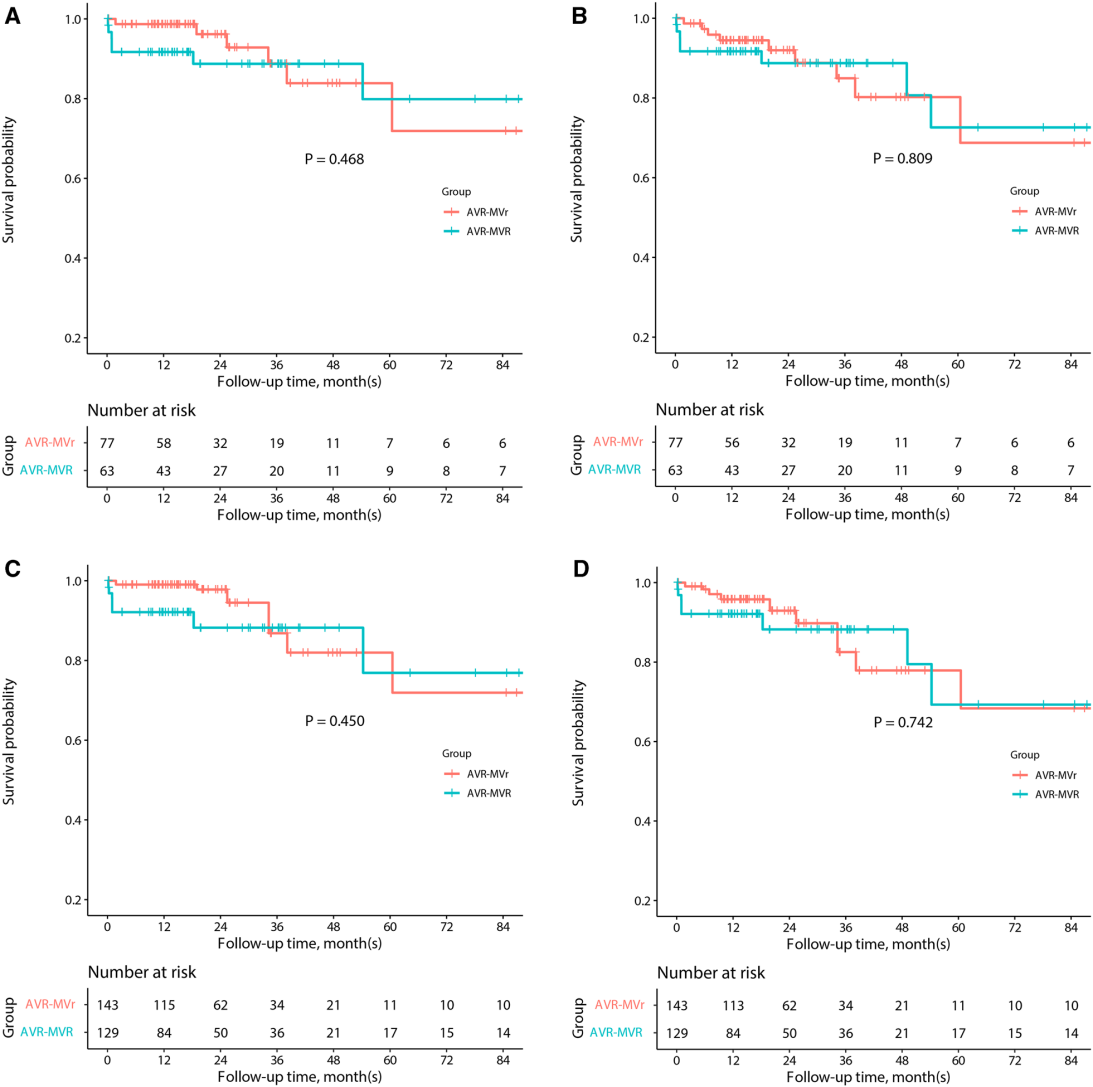
Kaplan–Meier estimates of survival outcomes in the subgroup of AVR-MVS patients. Overall survivals in the original cohort (**A**) and IPTW analysis (**C**), as well as MACCE-free survival in the original cohort (**B**) and IPTW analysis (**D**). AVR, aortic valve replacement; IPTW, inverse probability treatment weighting; MACCE, major adverse cardiovascular and cerebrovascular events; MVr, mitral valve repair; MVR, mitral valve replacement; MVS, mitral valve surgery

To further investigate the prognostic effect of MVS on the patients, AVR-MVS patients were divided into AVR-MVr and AVR-MVR groups. Baseline and operative characteristics were well-balanced (Additional file 1: Table S1). As compared to AVR-MVr, AVR-MVR increased the risk of operative mortality (0 vs. 7.9%, P = 0.012) and thoracotomy for bleeding (0 vs. 7.9%, P = 0.012), which was sustained in the IPTW analysis. However, follow-up mortality (P_log-rank_ = 0.468) and the rate of MACCE (P_log-rank_ = 0.809) were comparable between the two groups, and these results were maintained in the IPTW analysis (Fig. 2).

Considering the difference between the aortic regurgitation and stenosis patients, subgroup analysis was performed. In the subgroup of aortic stenosis, AVR-MVS did not reduce the risk of mortality (P_log-rank_ = 0.197) or MACCE (P_log-rank_ = 0.907) than isolated AVR. On the contrast, however, AVR-MVS increased the risk of mortality (P_log-rank_ = 0.048) but not MACCE (P_log-rank_ = 0.410) in the aortic regurgitation subgroup.

## Discussion

This study presents a comprehensive evaluation of MVS in patients with moderate FMR and HFpEF who are undergoing AVR. The primary finding is that AVR-MVS as compared with AVR displayed greater risk of operative and follow-up mortality, as well as a trend to increased risk of MACCE. Further investigations revealed that increased risk of operative mortality and postoperative thoracotomy for bleeding were attributed majorly to MVR rather than MVr, while the latter two techniques showed similar follow-up death and MACCE.

HFpEF is highly prevalent in the clinical practice, accounting for more than 50% percent of the heart failure patients [10]. Being one of the major etiologies, aortic valve disease is not uncommon in heart failure patients [12]. Studies report that aortic valve disease can be seen in a substantial proportion of HFpEF patients [13], and even low-grade aortic valve disease in these patients can increase the risk of mortality [14, 15]. Since it can have negative impact on the patient prognosis, the treatment of HFpEF, especially the etiological treatment, is critical. Unfortunately, however, limited studies are available.

The direct cause of FMR is due to the dysfunction or remodeling of left ventricle or left atrium. therefore, FMR is very common in patients with heart failure, even in HFpEF. Conventionally, mild to moderate FMR is considered to be an innocent bystander in patients with HFpEF. However, more and more studies demonstrate that FMR increases the risk of adverse events and compromises the quality of life in patients with HFpEF [16], and it is also found to be associated with increased risk of pulmonary hypertension [17, 18]. Researchers also report that even mild FMR is associated with increased adverse outcomes in HFpEF patients [3]. Nevertheless, no studies are available on the impact of FMR in HFpEF patients undergoing AVR. In this study, we noticed that moderate FMR has improved in HFpEF patients immediately after isolated AVR and persisted during the mid-term follow-up. Meanwhile, no death was observed during the perioperative and follow-up period after isolated AVR. The possible explanation is that both FMR and HFpEF is attributed to the left ventricular dysfunction caused by the aortic valve disease in this group of patients. And after correction of the aortic valve stenosis or insufficiency, the left ventricular dysfunction has been relieved, followed by the improvement HFpEF and FMR.

As mentioned previously, studies report that even mild FMR can increase the risk of adverse events. Therefore, researchers conclude in their study that FMR should be the focus of strategies attempting to reduce heart failure [2]. Studies have evaluated the effect of MVr in different entities of FMR. Balogh et al. report in their study [4] that patients with atrial FMR and HFpEF benefit from endoscopic MVr. Elsewhere, Stone et al. [5] notice that in patients with moderate-to-severe or severe FMR, transcatheter mitral valve repair, as compared to medical therapy, decreases the risk of mortality by 38% during a follow-up of 2 years. However, the studies included heart failure patients with reduced, mid-range and preserved ejection fraction. Nonetheless, there is no study evaluating the effect of MVS in patients with moderate FMR and HFpEF undergoing AVR. In this study, we observed that AVR-MVS was associated with increased risk of operative and follow-up mortality, as well as the postoperative thoracotomy for bleeding, when compared to the isolated AVR. Furthermore, in the subgroup analysis, we noticed that AVR-MVR increased the risk of operative death and postoperative thoracotomy for bleeding rather than AVR-MVr, while no significant difference was found regarding follow-up mortality and MACCE between the two subgroups. Therefore, isolated AVR might be more reasonable than AVR-MVS in this group of patients. More studies with larger sample sizes and prospective design is needed.

This study has several limitations. First of all, this was a retrospective study, and the potential bias caused by the observational design could not be avoided. Secondly, the sample size in this study was limited, restricting the power of tests. Furthermore, the surgeons’ judgement and preference might have also caused bias to some extent. In addition, although IPTW analysis has priority on balancing the baseline variables, unmeasured confounders may still exist. Last but by no means the least, follow-up echocardiographic results were not available for all of the patients survived during the study period, which may also have caused potential bias.

## Conclusions

In patients with moderate FMR and HFpEF, isolated AVR might be more reasonable than AVR-MVS.

## Supplementary Information

**Additional file 1. Table S1.** Comparison of baseline characteristics and outcomes of AVR-MVr and AVR-MVR.

## Data Availability

The datasets used and/or analysed during the current study are available from the corresponding author on reasonable request.

## Abbreviations

CI: Confidence interval
EF: Ejection fraction
FMR: Funcitonal mitral regurgitation
HR: Hazard ratio
LAD: Left atrial diameter
LVEDD: Left ventricular end-diastolic diameter
MACCE: Major adverse cardiovascular and cerebrovascular events
NT-proBNP: N-terminal pro-B type natriuretic peptide
AVR: Aortic valve replacement
HFpEF: Heart failure with preserved ejection fraction
IPTW: Inverse probability treatment weighting
MVr: Mitral valve repair
MVS: Mitral valve surgery
MVR: Mitral valve replacement
