# Supplementary material for: Impact of concomitant mitral valve surgery on the clinical outcomes of patients with moderate functional mitral regurgitation and HFpEF undergoing aortic valve replacement: a cohort study
Source: J Cardiothorac Surg. 2023 Apr 5;18:100. doi: 10.1186/s13019-023-02197-2 (PMC10077695; doi:10.1186/s13019-023-02197-2)
Supplement: Supplementary file 1 — Additional file 1. Table S1. Comparison of baseline characteristics and outcomes of AVR-MVr and AVR-MVR. [file 13019_2023_2197_MOESM1_ESM.docx]

**Table S1. Comparison of baseline characteristics and outcomes of AVR-MVr and AVR-MVR.**

| Variables | Original | | |  | IPTW analysis | | |
| --- | --- | --- | --- | --- | --- | --- | --- |
|  | AVR-MVr (N=77) | AVR-MVR (N=63) | P value |  | AVR-MVr (N=142.97) | AVR-MVR (N=129.21) | P value |
| ***Preoperative*** | | | | | | | |
| Age (years), mean ± SD | 57.1 ± 12.5 | 58.5 ± 12.34 | 0.511 |  | 58.0 ± 12.1 | 57.9 ± 12.5 | 0.941 |
| Female, no (%) | 16 (20.8) | 18 (28.6) | 0.285 |  | 38.7 (27.0) | 34.9 (27.0) | 0.998 |
| BMI (kg/m^2^), median [Q1, Q3] | 24.5 [21.5, 27.7] | 23.0 [21.4, 24.8] | 0.035 |  | 23.2 [20.8, 26.3] | 23.3 [21.8, 25.5] | 0.832 |
| BSA (m^2^), median [Q1, Q3] | 1.8 [1.7, 2.0] | 1.8 [1.6, 1.9] | 0.042 |  | 1.7 [1.6, 1.9] | 1.8 [1.7, 1.9] | 0.771 |
| Hypertension, no (%) | 35 (45.5) | 21 (33.3) | 0.145 |  | 59.2 (41.4) | 49.9 (38.7) | 0.762 |
| Dyslipidemia, no (%) | 29 (37.7) | 20 (31.7) | 0.465 |  | 51.0 (35.7) | 44.0 (34.0) | 0.855 |
| Smoking, no. (%) | 38 (49.4) | 24 (38.1) | 0.182 |  | 60.9 (42.6) | 55.6 (43.0) | 0.964 |
| Diabetes mellitus, no (%) | 6 (7.8) | 7 (11.1) | 0.501 |  | 13.1 (9.1) | 12.4 (9.6) | 0.936 |
| Coronary artery disease, no (%) | 18 (23.4) | 7 (11.1) | 0.059 |  | 24.5 (17.1) | 19.2 (14.9) | 0.756 |
| Atrial fibrillation, no (%) | 11 (14.3) | 12 (19.0) | 0.449 |  | 22.9 (16.0) | 20.4 (15.8) | 0.972 |
| Renal failure, no (%) | 5 (6.5) | 3 (4.8) | 0.661 |  | 7.4 (5.2) | 5.1 (3.9) | 0.707 |
| Stroke, no (%) | 5 (6.5) | 1 (1.6) | 0.154 |  | 5.7 (4.0) | 2.5 (1.9) | 0.483 |
| Aortic valve disease, no (%) | | | 0.563 |  |  | | 0.926 |
| Aortic insufficiency | 56 (72.7) | 43 (68.3) |  |  |  |  |  |
| Aortic stenosis | 21 (27.3) | 20 (31.7) |  |  | 44.1 (30.9) | 40.9 (31.7) |  |
| NYHA III/IV, no (%) | 42 (54.5) | 32 (50.8) | 0.658 |  | 77.0 (53.9) | 67.1 (51.9) | 0.833 |
| LAD (mm), mean ± SD | 46.6 ± 6.8 | 46.1 ± 5.7 | 0.632 |  | 46.2 ± 6.5 | 45.9 ± 5.4 | 0.784 |
| LVEDD (mm), mean ± SD | 65.3 ± 9.3 | 64.3 ± 8.6 | 0.509 |  | 64.4 ± 9.6 | 64.1 ± 8.5 | 0.831 |
| EF (%), mean ± SD | 58.7 ± 5.1 | 57.5 ± 4.2 | 0.140 |  | 58.0 ± 5.1 | 57.7 ± 4.4 | 0.710 |
| NT-proBNP (pg/ml), median [Q1, Q3] | 1287.0 [638.2, 2479.7] | 1526.0 [802.9, 2456.4] | 0.553 |  | 1305.6 [732.8, 2410.1] | 1401.0 [791.8, 2437.1] | 0.754 |
| ***Operative*** | | | | | | | |
| Concomitant procedures |  |  |  |  |  |  |  |
| CABG, no (%) | 13 (16.9) | 6 (9.5) | 0.206 |  | 18.6 (13.0) | 16.3 (12.6) | 0.954 |
| TV repair, no (%) | 17 (22.1) | 27 (42.9) | 0.008 |  | 48.4 (33.9) | 44.4 (34.4) | 0.957 |
| Other procedures, no (%) | 10 (13.0) | 4 (6.3) | 0.193 |  | 14.6 (10.2) | 11.9 (9.2) | 0.864 |
| CPB (min), median [Q1, Q3] | 146.0 [124.0, 183.0] | 143.0 [123.0, 182.5] | 0.815 |  | 140.3 [121.7, 176.4] | 139.7 [119.0, 181.9] | 0.962 |
| Cross-clamp (min), median [Q1, Q3] | 113.0 [92.0, 136.0] | 112.0 [93.0, 144.0] | 0.683 |  | 110.3 [92.0, 132.4] | 111.7 [89.1, 144.0] | 0.822 |
| ***Early postoperative*** |  |  |  |  |  |  |  |
| Perioperative IABP, no (%) | 0 | 1 (1.6) | 0.267 |  | 0 | 4.3 (3.3) | 0.280 |
| Perioperative transfusion, no (%) | 6 (7.8) | 10 (15.9) | 0.135 |  | 8.0 (5.6) | 21.3 (16.5) | 0.032 |
| New-onset stroke, no (%) | 0 | 1 (1.6) | 0.267 |  | 0 | 2.1 (1.6) | 0.294 |
| New-onset atrial fibrillation, no (%) | 5 (6.5) | 4 (6.3) | 0.972 |  | 8.3 (5.8) | 6.9 (5.3) | 0.907 |
| Acute kidney injury, no (%) | 6 (7.8) | 5 (7.9) | 0.975 |  | 13.1 (9.2) | 12.1 (9.4) | 0.978 |
| Thoracotomy for bleeding, no (%) | 0 | 5 (7.9) | 0.012 |  | 0 | 8.0 (6.2) | 0.027 |
| Operative death, no (%) | 0 | 5 (7.9) | 0.012 |  | 0 | 9.5 (7.3) | 0.023 |
| LAD (mm), mean ± SD | 38.5 ± 5.4 | 40.0 ± 5.9 | 0.128 |  | 38.0 ± 5.1 | 39.8 ± 6.0 | 0.065 |
| Δ LAD (mm), mean ± SD | -8.1 ± 5.8 | -6.1 ± 6.8 | 0.066 |  | -8.2 ± 6.0 | -6.1 ± 6.7 | 0.071 |
| LVEDD (mm), mean ± SD | 53.9 ± 7.0 | 53.1 ± 7.5 | 0.500 |  | 53.4 ± 6.6 | 52.4 ± 7.9 | 0.488 |
| Δ LVEDD (mm), mean ± SD | -11.4± 7.8 | -11.2 ± 7.2 | 0.887 |  | -11.1 ± 7.8) | -11.7 ± 6.7 | 0.649 |
| EF (%), mean ± SD | 52.1 ± (9.4) | 52.8 ± (6.8) | 0.625 |  | 51.8 ± (9.7) | 53.2 ± (6.6) | 0.370 |
| Δ EF (%), mean ± SD | -6.6 ± (8.3) | -4.7 ± (7.6) | 0.164 |  | -6.2 ± (8.1) | -4.5 ± (7.9) | 0.259 |
| Mitral regurgitation, no (%) | | | < 0.001 |  |  | | <0.001 |
| No | 48 (62.3) | 60 (95.2) |  |  | 88.7 (62.1) | 123.9 (95.9) |  |
| Trivial | 26 (33.8) | 3 (4.8) |  |  | 50.2 (35.1) | 5.3 (4.1) |  |
| mild | 3 (3.9) | 0 |  |  | 4.1 (2.9) | 0 |  |
| Improvement of FMR, no (%) | 77 (100.0) | 63 (100.0) | > 0.99 |  | 142.9 (100.0) | 129.2 (100.0) | > 0.99 |
| ***Follow-up*** | | | | | | | |
| All-cause death, no (%) | 6 (7.8) | 8 (12.7) | 0.468 ^*^ |  | 11.0 (7.7) | 16.3 (12.6) | 0.450 ^*^ |
| MACCE, no (%) | 9 (11.7) | 9 (14.3) | 0.809 ^*^ |  | 16.7 (11.7) | 18.4 (14.2) | 0.742 ^*^ |

* Log-rank test.

AVR, aortic valve replacement; BMI, body mass index; BSA, body surface area; CABG, coronary artery bypass grafting; CPB, cardiopulmonary bypass; EF, ejection fraction; IABP, intra-aortic balloon pump; IPTW, inverse probability treatment weighting; LAD, left atrial diameter; LVEDD, left ventricular end-diastolic diameter; MACCE, major adverse cardiovascular and cerebrovascular events; MVr, mitral valve repair; MVR, mitral valve replacement; NT-proBNP, N-terminal pro-B type natriuretic peptide; NYHA, New York Heart Association; SD, standard deviation; TV, tricuspid valve.
